# The burden and natural history of cardiac pathology at TB diagnosis in a high-HIV prevalence district in Zambia: protocol for the TB-Heart study

**DOI:** 10.1186/s12872-024-03877-0

**Published:** 2024-04-10

**Authors:** Marcello S. Scopazzini, Pamela Chansa, Edith D. Majonga, Nina Bual, Albertus Schaap, Kondwelani J. Mateyo, Remmy Musukuma, Veronica Mweemba, Maina Cheeba, Chipili C. Mwila, Lucheka Sigande, Isabel Banda, Joseph Ngulube, Kwame Shanaube, Dominik Zenner, Helen Ayles, Anoop S. V. Shah

**Affiliations:** 1 Department of Non-Communicable Diseases Epidemiology, Faculty of Epidemiology and Population Health, London School of Hygiene and Tropical Medicine, Keppel Street, London, WC1E 7HT UK; 2 Zambart, University of Zambia - Ridgeway Campus, Off-Nationalist Road, Lusaka, Zambia; 3 Department of Cardiology, University Teaching Hospital, Nationalist Road, Lusaka, Zambia; 4 Biomedical Research Training Institute, 10 Seagrave, Avondale, Harare, Zimbabwe; 5 Department of Oncology, Medical Physics and Imaging Sciences, Faculty of Medicine and Health Sciences, University of Zimbabwe, Harare, Zimbabwe; 6 Echocardiography Department, St Mary’s Hospital, Praed Street, London, W2 1NY UK; 7 Department of Infectious Diseases Epidemiology, London School of Hygiene and Tropical Medicine, Keppel Street, London, WC1E 7HT UK; 8 Department of Clinical Research, Faculty of Infectious and Tropical Diseases, London School of Hygiene and Tropical Medicine, Keppel Street, London, WC1E 7HT UK; 9 Wolfson Institute of Population Health, Mary University of London, London, Queen UK

**Keywords:** Pulmonary tuberculosis, Cardiovascular disease, Non-communicable diseases, Echocardiography, Cardiac biomarkers

## Abstract

**Background:**

Tuberculosis (TB) continues to be a major cause of death across sub-Saharan Africa (SSA). In parallel, non-communicable disease and especially cardiovascular disease (CVD) burden has increased substantially in the region. Cardiac manifestations of TB are well-recognised but the extent to which they co-exist with pulmonary TB (PTB) has not been systematically evaluated. The aim of this study is to improve understanding of the burden of cardiac pathology in PTB in those living with and without HIV in a high-burden setting.

**Methods:**

This is a cross-sectional and natural history study to evaluate the burden and natural history of cardiac pathology in participants with PTB in Lusaka, Zambia, a high burden setting for TB and HIV. Participants with PTB, with and without HIV will be consecutively recruited alongside age- and sex-matched TB-uninfected comparators on a 2:1 basis. Participants will undergo baseline assessments to collect clinical, socio-demographic, functional, laboratory and TB disease impact data followed by point-of-care and standard echocardiography. Participants with PTB will undergo further repeat clinical and functional examination at two- and six months follow-up. Those with cardiac pathology at baseline will undergo repeat echocardiography at six months.

**Discussion:**

The outcomes of the study are to a) determine the burden of cardiac pathology at TB diagnosis, b) describe its association with patient-defining risk factors and biochemical markers of cardiac injury and stretch and c) describe the natural history of cardiac pathology during the course of TB treatment.

## Background

Tuberculosis (TB) and Human Immunodeficiency Virus (HIV) infection continue to be major public health concerns in sub-Saharan Africa (SSA) and in Zambia. In parallel, non-communicable diseases (NCD) burden is increasing and cardiovascular diseases (CVD) now account for 1 in 10 deaths [1–3]. A recent meta-analysis found a 1.5 greater risk of cardiovascular disease (CVD) mortality in patients with TB disease but most data on TB-associated CVD originate from non-endemic regions in high-income settings (HICs) where atherosclerotic CVD predominates [4]. In contrast, mechanisms contributing to CVD burden in SSA are believed to be non-ischaemic in aetiology and consist primarily of pericardial and cardiomyopathic pathology [4–8].

TB is a chronic bacterial infection caused by *Mycobacterium tuberculosis* that commonly affects the lung (pulmonary TB [PTB]) [9]. Cardiac manifestations occur secondary to lymphatic spread typically affecting the right side of the heart and the pericardium [10, 11]. TB pericarditis accounts for up to 50% of all constrictive pericarditis and significantly contributes to heart failure and cardiovascular burden in SSA [10].

Zambia is one of 30 high-burden countries for TB and HIV, with an estimated TB incidence of 330 per 100,000 and an 11% prevalence of HIV in the general population [1, 12]. Approximately 50% of patients with newly diagnosed TB are living with HIV. The age-standardised total CVD death rate is approximately 10% [13]. Recent post-mortem studies in Zambia have reported evidence of cardiac involvement at autopsy in 4–7% of TB deaths [14, 15]. This suggests that dissemination to cardiac structures in TB disease remains underappreciated.

People living with HIV (PLHIV) who develop TB disease are at higher risk of extra-pulmonary manifestations, dissemination and early mortality [16, 17]. Overt and subclinical cardiac pathology is common in HIV with dilated cardiomyopathy (DCM) and pericardial diseases accounting for 38% and 12.5% respectively of all CVD admissions in PLHIV in a SSA setting [18–21]. Systematic evaluation of pericardial and/or cardiomyopathic involvement in patients with PTB with and without HIV – especially in the anti-retroviral therapy (ART)-era – is lacking. Further, how cardiac involvement evident at PTB diagnosis progresses during the course of TB treatment, or whether PTB contributes to future CVD burden in SSA, remains unknown.

The overarching aim of the study is to understand the burden and natural history of cardiac disease in people with pulmonary tuberculosis.

## Methods / design

This is a cross-sectional and natural history study in which we seek to 1) estimate the burden of cardiac disease in 250 consecutive participants newly diagnosed with PTB and 2) explore the natural history of cardiac pathology in patients with PTB over the course of standard TB treatment, expected to last approximately six months.

Participants will be recruited at a large district-level hospital in Lusaka, Zambia, that serves a a high-density township with high levels of poverty and has an estimated TB incidence of 850/100,000 in patients aged > 15 and an HIV prevalence of 20% among adults aged 18–44 [Zambart, unpublished data, 2020]. We expect approximately 50% of our participants to be PLHIV.

We aim to recruit age- and sex-matched comparators on a 2:1 ratio, or approximately 125 comparators. Comparators will be participants living with and without HIV where TB disease has been excluded and will provide an estimate of prevalent cardiac pathology in the general outpatient population attending Kanyama Hospital.

### Selection criteria for PTB patients

Participants with PTB will be identified by the study team onsite at the TB clinic at Kanyama Hospital, where all newly diagnosed patients are enrolled for treatment initiation. All patients who meet inclusion criteria will be eligible for recruitment and will be appropriately sensitised prior to obtaining written informed consent for participation in the study.

#### Inclusion criteria

Participants with PTB must be aged ≥ 18, provide signed informed consent (or witnessed thumb-printed informed consent if illiterate) for study participation, and present with a new diagnosis of PTB and/or PTB with extrapulmonary TB (EPTB) defined as a) bacteriologically confirmed disease on sputum, positive by Xpert MTB/RIF® (Cepheid, Sunnyvale, CA, USA) polymerase chain reaction (PCR) test and/or b) radiologically-confirmed disease within the preceding two weeks. 

#### Exclusion criteria

Participants will be excluded if they 1) decline or are unable to provide consent, 2) if they are current hospital inpatients, 3) if they are pregnant, 4) if they have tuberculosis disease without pulmonary involvement 5) if they are diagnosed with Rifampicin-resistant TB.

### Selection criteria for comparator population

HIV-negative comparator participants will be approached within the healthcare facility whilst comparator participants who are PLHIV will be individuals established on anti-retroviral therapy (ART) for more than 6 months recruited from HIV outpatient clinics.

Comparator participants must be aged ≥ 18, able to provide signed informed consent (or witnessed and thumb-printed informed consent if illiterate) and have a documented negative World Health Organization (WHO) four symptom screen for TB (WHO 4SS) and a negative Xpert MTB/RIF® PCR test. Comparator participants living with HIV must have been established on anti-retroviral therapy (ART) for longer than six months with documented HIV viral load suppression.

Comparator participants will be excluded if they 1) refuse or are unable to provide informed consent, 2) have one or more clinical symptoms of TB during WHO 4SS, 3) are clinically unwell at the time of recruitment, 4) are hospital inpatients, 5) have a documented history of cardiovascular disease, 6) have not been established on ART for > 6 months, or 7) refuse or are unable to provide sputum for Xpert MTB/RIF® PCR.

### Study activities

Recruitment to the TB-HEART studies commenced in November 2023. Participants with PTB will be followed up longitudinally until they complete TB treatment at six months in accordance with Zambian TB treatment guidelines. The natural history study is expected to be completed in February 2025, or six months after the last participant with PTB is recruited to the cross-sectional study.

**Fig. 1:**
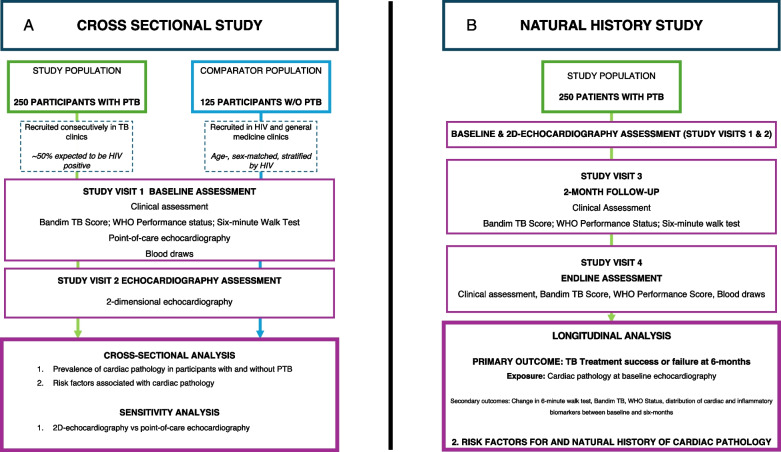
**A** Study Diagram: Cross-sectional Study to determine the prevalence of cardiac pathology in consecutively recruited participants with PTB in Lusaka, Zambia. 250 consecutively recruited participants with PTB will be matched to comparator participants without PTB on a 2:1 basis. Participants and comparators will undergo a comprehensive clinical assessment; functional assessments including Bandim TB score, WHO Performance status and six-minute walk test; point-of-care echocardiography; and 2D-echocardiography. **B** Study Diagram: Natural history study where participants with PTB will be followed up at 2- and 6-months to a) evaluate TB outcomes and b) determine change in functional status over time and c) biomarkers of inflammation and cardiac pathology in those with PTB with and without echocardiographic evidence of cardiac pathology at diagnosis. Legend: 6MWT = 6-min walk test

The study procedures for the cross-sectional prevalence study and natural history study are described in Fig. 1A and B.

### Clinical and functional assessments

We will use study-specific electronic questionnaires to capture the following data at enrolment and subsequent study visits: 1) sociodemographic details including age, sex, household income, address and contact details 2) focused medical and drug history to capture type and duration of TB symptoms; past medical and cardiovascular disease history and, where applicable, HIV diagnosis and treatment history 3) clinical observations including blood pressure, heart rate, respiratory rate, pulse oximetry, and temperature and 4) detailed cardiac, respiratory and abdominal examination.

Functional assessment of TB disease severity at baseline and follow-up visits will be assessed using 1) the Bandim TB score, a standardised questionnaire to identify patients at highest risk of treatment failure and/or death following TB diagnosis [22] 2) WHO Performance status and 3) six-minute walk test (6MWT), a standardised protocol to assess functional respiratory capacity [23].

### Cardiac ultrasound

#### Point of care echocardiography

A study-specific protocol adapted from the Society of Intensive Care Medicine Focused Ultrasound for Intensive Care (FUSIC) Heart protocol will be used to rapidly assess cardiac anatomy and function including presence or absence of pericardial disease and/or left ventricular systolic dysfunction in all participants at their baseline visit using a Clarius® phased-array handheld cardiac ultrasound probe (Clarius Mobile Health, Vancouver, BC, Canada) [24].

Diagnostic images will be stored securely on a password-protected tablet linked to the handheld probe. Point-of-care echocardiography results will be shared with the patient and their treating clinician in Zambia to ensure appropriate management according to local clinical guidelines, if necessary.

#### Standard echocardiography

All participants will undergo two-dimensional (2D) echocardiography which will capture images in parasternal long- and short-axis; four-chamber; two-chamber; five-chamber; and subcostal views followed by doppler assessment and assessment for pericardial pathology.

Echocardiography assessments will take place at the Echocardiography Department at University Teaching Hospital and are performed by trained echocardiographers using a GE Logiq S7 Expert Ultrasound Machine® (General Electric, Boston, MA, USA). The minimum image acquisition dataset adheres to British and European Society for Echocardiography standards.

Participants with PTB will be given appointments at least two weeks after starting anti-tuberculous therapy in compliance with infection control procedures to minimise cross-infection risk.

All participants with echocardiographic evidence of cardiac pathology will be referred for further assessment in line with local guidelines and will be offered a repeat echocardiogram following completion of TB treatment.

#### Image acquisition, storage, and analysis

Images will be captured in Digital Imaging and Communications in Medicine (DICOM®) format, anonymised, and stored on an encrypted and password protected study computer and secure cloud-based server.

Echocardiography assessments will be reported in real-time and results shared with the participant and their responsible clinical team. To minimise reporter bias, a proportion of participant DICOM® cine loops and images will be externally reviewed by expert members of the study team.

### Blood sample testing including cardiac and inflammatory biomarkers

The study team will collect up to 10 millilitres (ml) whole blood in Ethylene-Diamene-Tetra-Acetic (EDTA) from each participant at baseline and endline assessments ensuring appropriate aseptic non-touch technique.

Blood samples will be tested for: 1) C-reactive protein (CRP) on the Abbott Afinion® point-of-care analyser (Abbott Laboratories, Abbott Park, IL, USA); and 2) high-sensitivity cardiac troponin I (hs-cTnI) and N-terminal pro B-type natriuretic peptide (NT pro-BNP) on Abbott Architect® (Abbott Laboratories, Abbott Park, IL, USA). Remaining sera will be aliquoted and stored in -80 degree freezers.

We will offer HIV testing to any participant who has not undergone an HIV test in the preceding six months, unless the participant is already living with HIV. If the test is reactive, the participant will be referred to the local HIV team.

All blood samples of participants who are PLHIV will be tested for CD4 count on the PIMA™ CD4 point-of-care analyser (Abbott Laboratories, Abbott Park, IL, USA) and HIV viral load on the Xpert® HIV-1 Viral Load point-of-care analyser (Cepheid, Sunnyvale, CA, USA).

### Study visits

#### Baseline assessment

The baseline assessment comprises of two study visits: study visit 1 is the comprehensive clinical and functional assessment where we capture anthropometric and socio-economic data; standardised clinical observation and clinical examination findings; 12-lead electrocardiogram; functional assessment including Bandim TB score, WHO performance status and 6MWT; point-of-care echocardiography; and blood sampling.

Participants are then invited to study visit 2 to undergo 2D-echocardiography at University Teaching Hospital. Comparator participants are not routinely followed-up beyond this visit, however, any participant where abnormalities are found on echocardiography is referred for further investigation and management with the local cardiology team.

#### Two- and six-month follow up assessments

All participants with PTB will be followed-up at two months – study visit 3, to coincide with expected step-down from intensive to continuation phase anti-tuberculous treatment – when the study team will repeat clinical and functional assessments only.

Participants with PTB will be reviewed at approximately six months to coincide with the expected completion of TB treatment as per local standard of care – study visit 4. Clinical and functional assessments and blood sampling will be repeated. We will record TB treatment outcomes in line with standard WHO definitions and all participants with PTB will be invited to undergo a repeat 2D-echocardiography assessment at UTH.

**Fig. 2:**
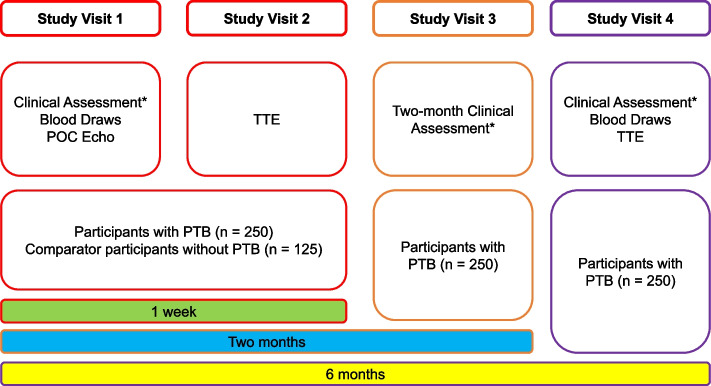
Study Flow Diagram. Describes the study visits that participants will attend over the course of six months from enrolment. *Clinical assessment is history and examination, functional assessment (Bandim TB, WHO Performance Status, and six-minute walk test). Blood draws are cardiac biomarkers including high-sensitivity troponin I and non-terminal pro-B type natriuretic peptide, and inflammatory biomarkers including C-reactive protein. POC Echo = point-of-care echocardiography; TTE = transthoracic echocardiogram; PTB = pulmonary TB

Study visits are summarised in Fig. 2 – Study Visits flow diagram.

#### Long-term follow-up

Participants with PTB will be followed up until they complete TB treatment, or approximately six months following recruitment.

No further follow-up visits are planned beyond six months, but we plan to keep a record of all participants recruited to the study to enable the study team to evaluate longer-term health consequences of TB disease and cardiovascular health in the future.

#### Loss to follow-up

Detailed participant locator forms listing two contact numbers will be completed where possible, and participants will be encouraged to contact the study team at any point during the study if necessary.

Participants will be provided with a two- and six-month appointment date and will receive a reminder telephone call from the research team to confirm the validity of demographic information and enquire as to the participant’s ongoing consent to remain in the study.

Participants who remain uncontactable after two months will receive a home visit. If no further contact can be made beyond this time, the participant will be recorded as lost to follow-up.

## Objectives

We have six objectives:

1. To determine the prevalence of echocardiographic evidence of cardiac pathology in participants with PTB compared to those without PTB in Zambia
2. To identify risk factors associated with echocardiographic evidence of cardiac pathology in participants with PTB compared to those without PTB in Zambia
3. To explore the diagnostic accuracy of point-of-care echocardiography alone or in combination with biochemical markers of cardiac injury and/or stretch compared to standard echocardiography to detect evidence of cardiac pathology in participants with PTB.
4. To compare a) World Health Organization (WHO) classification of clinical outcomes of PTB (including death or treatment success) at six months, and b) improvement in Bandim TB, WHO Performance Score, and six-minute walk test at diagnosis, two months, and six months, in participants with PTB with and without evidence of cardiac pathology at diagnosis
5. To describe a) the distribution of circulating biomarkers of inflammation and cardiac pathology and b) explore their association with echocardiographic evidence of cardiac pathology in participants with PTB at diagnosis and at six months
6. To explore the natural history of echocardiographic evidence of cardiac pathology in participants with PTB.

## Study outcomes

### Cross-sectional Study

The primary outcome is the prevalence of echocardiographic evidence of cardiac pathology identified at 2D-echocardiography assessment in participants with PTB compared to matched comparators without evidence of PTB.

Cardiac pathology is defined as:

- presence of pericardial disease (pericardial effusion > 0.5 cm or pericardial thickening > 2 mm)
  and/or
- left ventricular systolic dysfunction (left ventricular ejection fraction < 50% by Simpson’s Biplane measurement).

Secondary outcomes include: 1) the association between echocardiographic evidence of cardiac pathology and relevant socio-demographic and patient characteristics, and risk factors for cardiac pathology, including HIV status; and 2) sensitivity and specificity of point-of-care echocardiography in detecting cardiac pathology alone or in combination with biochemical markers of cardiac pathology compared to standard echocardiography.

### Natural history study

The primary outcome is death and/or treatment success at completion of TB treatment among participants with PTB with and without cardiac pathology at baseline assessment.

Secondary outcomes include: 1) change in six-minute walk test, Bandim TB Score, WHO Performance Status between baseline, two month and six month follow-up assessments 2) distribution of circulating biochemical markers of inflammation (CRP), myocardial injury (hs-cTnI) and stretch (NT pro-BNP) and their association with echocardiographic evidence of cardiac pathology in participants with PTB with and without cardiac pathology at baseline assessment and 3) prevalence and distribution of echocardiographic and biochemical evidence of cardiac pathology in participants with PTB and cardiac pathology at baseline and at six months or completion of PTB treatment.

### Sample size

The primary outcome measure used to determine sample size was the absolute difference in prevalence of cardiac pathology between participants with and without PTB, stratified by HIV status.

A consecutive case series in Malawi showed that 27% of patients with PTB, of whom 78% were living with HIV, had evidence of pericardial involvement on point-of-care ultrasound [Scopazzini, unpublished data]. The prevalence of cardiac pathology in patients with PTB who are HIV-negative has not been systematically evaluated and is therefore unknown.

We conservatively assumed a prevalence of 15% cardiac pathology in PTB patients. A sample size of 250 participants will provide us a precision estimate of approximately ± 4.6% with a 95% exact binomial confidence interval of 10.4%-19.6%.

Assuming a 15% prevalence of cardiac pathology in the PTB group (250 participants) and 5% in the non-PTB group (125 participants), the study would have 85% power to detect such a difference in prevalence.

Restricted to PTB patients only, assuming a 22% prevalence of cardiac pathology in the HIV-infected PTB patients and 8% in HIV-uninfected PTB patients, the study would have 88% power to detect such a difference in prevalence of cardiac pathology.

For the natural history study, Assuming a prevalence of cardiac pathology of up to 27% in participants with PTB living with HIV, we expect to follow-up up to 30 participants with PTB living with HIV and up to 15 HIV-uninfected participants with PTB. Participants with PTB and cardiac pathology will be matched to equal numbers of age and sex matched participants without cardiac pathology within the cohort.

### Analysis approach

Descriptive statistics for baseline clinical, imaging, and biomarker data will be provided across the entire group and stratified by presence or absence of PTB, with and without HIV infection. Estimates of the prevalence of echocardiographic evidence of cardiac pathology among people with and without PTB will be presented in addition to the prevalence estimates in the PTB patients stratified by HIV status. Prevalence odds ratio (POR) of participants with echocardiographic evidence of cardiac pathology comparing those with and without PTB will be presented across the study population and stratified by HIV status.

Across PTB patients, adjusted logistic regression models will be constructed to explore the association between echocardiographic-based cardiac pathology and a) biomarkers of cardiac pathology and b) risk factors for cardiac disease including HIV, age, gender, duration of PTB symptoms and PTB disease severity, pre-existing cardiovascular disease, and prior history of TB disease.

Sensitivity and specificity of point-of-care echocardiography will be tested against the gold standard of standard echocardiography and will assess point-of-care echocardiography alone and point-of-care echocardiography in combination with biomarkers of cardiac disease.

For the exploratory longitudinal analysis in our natural history study, we will construct a linear regression model to determine measures of association between outcomes of death (primary) and change in 6MWT (secondary), and exposures (cardiac pathology at baseline assessment) adjusting for time at TB treatment initiation as a fixed effect.

## Discussion

In this study, we seek to systematically evaluate the prevalence of cardiac pathology among consecutive participants with newly-diagnosed PTB, and its possible associations with important patient-defining risk factors in a high-burden setting for TB and HIV.

Evidence describing cardiac pathology in PTB has important limitations: many studies are case reports or series with small sample sizes; and others are limited by significant selection bias where cardiac pathology is described in PTB patients with incident cardiac disease or in well-defined subgroups, such as those with post-TB lung disease [25–31].

To our knowledge, this is the first observational study to systematically establish the prevalence of and associations between cardiac pathology and newly-diagnosed PTB. We will use a range of modalities including state-of-the-art biochemical profiling of cardiac injury and stretch supplemented with non-invasive imaging of the myocardium by ultrasound [6, 19, 32–34]. Our study also evaluates the role of point-of-care cardiac ultrasound – with particular relevance to low resource settings – to demonstrate its potential as a screening tool for cardiac pathology among participants with PTB [35].

We expect that our initial findings may result in future studies exploring 1) mechanistic relationships between PTB and cardiac pathology using advanced cardiac imaging techniques such as cardiac magnetic resonance imaging (CMR) and positron-emission tomography (PET), 2) exploratory immunology studies to provide further evidence as to whether a dose-dependent relationship exists between prevalent cardiac pathology and PTB disease, 3) observational cohort studies to evaluate the impact of prevalent cardiac pathology on short and long-term outcomes in TB disease and future cardiovascular disease risk, and 4) intervention studies to explore the added value of anti-inflammatory medication to reduce the burden of TB-associated cardiac sequelae.

Our study has some limitations: our cross-sectional study design inherently limits our ability to comment on causal mechanisms underpinning the relationship between PTB and cardiac pathology; logistical hurdles precluded a larger sample size defined on expected cardiovascular outcomes. Next, our short follow-up period precludes us from exploring the relationship between PTB and atherosclerotic cardiovascular disease and limits our projected analyses to exploring its relationship with pericardial and myocardial disease only. Finally, our comparator population may suffer a degree of selection bias, thereby hampering our ability to adjust for important confounders of cardiac disease in our study population.

We hope nonetheless to provide novel insights into the relationship between cardiac pathology and pulmonary tuberculosis, the former a rapidly rising non-communicable disease, and the latter a continuing scourge compounded by high HIV prevalence in resource-restricted settings in sub-Saharan Africa.

## Data Availability

No datasets were generated or analysed during the current study.
